# Does climate change influence the spread of malaria in Benin? Insights from ecological niche modeling for surveillance efforts

**DOI:** 10.1093/inthealth/ihaf064

**Published:** 2025-06-09

**Authors:** Donald Romaric Yehouenou Tessi, Eben-Ezer Apelete, Sunday Berlioz Kakpo, Romeo Thierry Yehouenou Tessi, Aysel Çağlan Günal

**Affiliations:** Department of Environmental Sciences, Gazi University, Ankara 06560, Turkey; SOS Biodiversity NGO, Abomey-Calavi 10 BP 542, Benin; Laboratory of Forest Sciences, University of Abomey-Calavi, Benin; SOS Biodiversity NGO, Abomey-Calavi 10 BP 542, Benin; Laboratory of Forest Sciences, University of Abomey-Calavi, Benin; National University Hospital Center Hubert K Maga/Military Medical Hospital Center Cotonou (Benin), Cotonou 01 BP 386, Benin; Department of Environmental Sciences, Gazi University, Ankara 06560, Turkey; Biology Education Department, Faculty of Gazi Education, Gazi University, Ankara 06560, Turkey

**Keywords:** *Anopheles gambiae*, Benin, climate change, ecological niche modeling, malaria

## Abstract

**Background:**

Malaria is a severe and endemic disease, remaining one of the most prevalent tropical illnesses and a leading cause of death among children aged <5 y. *Anopheles gambiae*, the primary vector of malaria in Benin, plays a critical role in its transmission. This study aims to contribute to the health protection of populations in Benin by assessing the risk of vector-borne diseases, particularly malaria, in the context of climate change.

**Methods:**

Using the Maxent algorithm for ecological niche modeling, we mapped the distribution of *A. gambiae*, a highly effective vector of Plasmodium parasites.

**Results:**

Our findings revealed that high-risk areas for malaria cover nearly all departments of Benin, with the majority of southern departments—Mono, Littoral, Couffo, Ouémé, Plateau and Zou—identified as high-risk zones. Projections for 2055 under Representative Concentration Pathway (RCP) 4.5 and RCP 8.5 climate scenarios indicate a significant expansion of high-risk areas, extending to Collines and parts of Donga, Borgou and Atacora.

**Conclusions:**

Climate change is expected to exacerbate the spread of *A. gambiae*, increasing the disease risk across the country. These results are crucial for guiding policymakers in Benin to mitigate the current impact of malaria and implement preventative measures to address future risks.

## Introduction

Malaria, a vector-borne disease caused by the Plasmodium parasite and transmitted through the bites of infected female Anopheles mosquitoes, continues to pose a challenge to global health.^1^ It remains one of the deadliest infectious diseases in the world after lower respiratory tract infections, diarrheal diseases, HIV/AIDS and TB, especially in countries of tropical regions around the world.^2^ In 2023, >219 million cases of malaria, including 435 000 deaths, were reported in 87 countries.^3^ Benin, situated in West Africa, stands at the intersection of complex environmental, social and climatic factors that influence the prevalence and transmission dynamics of malaria. In fact, it is one of the 15 countries with the highest rate of malaria cases and deaths from the disease.^3^ In 2021, the incidence of malaria in the country was 21.2% for the general population and 48.1% among children aged <5 y.^4^ The 2024 malaria data for Benin underscore the country's struggle, with a reported 5 million cases emphasizing the need for sustained efforts and resources to address this public health concern.^5,6^

Mosquitoes, as vectors of various diseases, including malaria, contribute to several deaths each year through disease transmission. They constitute the largest family of vectors of pathogens that cause various public health diseases, including dengue, malaria, filariasis encephalitis, chikungunya fever, West Nile virus and Zika virus.^6^ Most of the main vectors of these diseases are *Aedes, Anopheles* and *Culex* genus mosquitoes, which are of major interest in the study of vector-borne diseases.^7^

*Anopheles gambiae* is considered as the main vector of malaria and lymphatic filariasis.^8,9^ As one of the most efficient vectors of the Plasmodium parasites, it is responsible for a substantial portion of malaria cases in sub-Saharan Africa and in Benin.^10–13^ Its preference for human blood and its adaptability to various environmental conditions make this mosquito species highly effective in spreading the malaria parasite.

Mosquito-borne diseases are climate-sensitive and climatic conditions set the geographic limits and seasonality of transmission.^14,15^ Climate change refers to long-term shifts in temperatures and weather patterns, primarily driven by human activities such as burning fossil fuels, deforestation and industrial processes.^16^ It is widely regarded as the most significant threat to global health, placing immense strain on healthcare systems and exacerbating existing health challenges.^17^ In fact, the dynamic nature of climate change introduces shifts in temperature, precipitation and other climatic parameters. These changes that can be expected under different climate change scenarios will affect the biology and ecology of vectors and intermediate hosts. Because of the disease burden associated with these mosquito species, there is a need to effectively monitor their populations.^18,19^ Understanding how these changes may affect the ecological niches of malaria vectors is essential for designing targeted and effective surveillance strategies. To unravel this complex relationship (climate change–malaria disease spread in Benin), a sophisticated tool that integrates environmental variables to predict the spatial distribution of species, such as ecological niche modeling (ENM), can be employed.^20^

In Benin, malaria remains one of the most significant public health threats, particularly in rural and peri-urban regions. Despite substantial control efforts, the disease burden persists, driven in part by environmental and climatic conditions that support year-round transmission. Understanding how climate variables influence the distribution of malaria vectors is crucial for predicting shifts in risk and guiding interventions, especially in the face of climate change.

This study aims to model the current and future distribution of *A. gambiae* in Benin using ENM. By integrating occurrence records with climate and land cover data, we project how vector suitability may change under different climate scenarios (Representative Concentration Pathway (RCP) 4.5 and RCP 8.5). Our findings are intended to support national malaria surveillance efforts and inform spatially targeted vector control strategies in anticipation of changing environmental conditions.

## Materials and methods

### Study area

**Figure 1.**
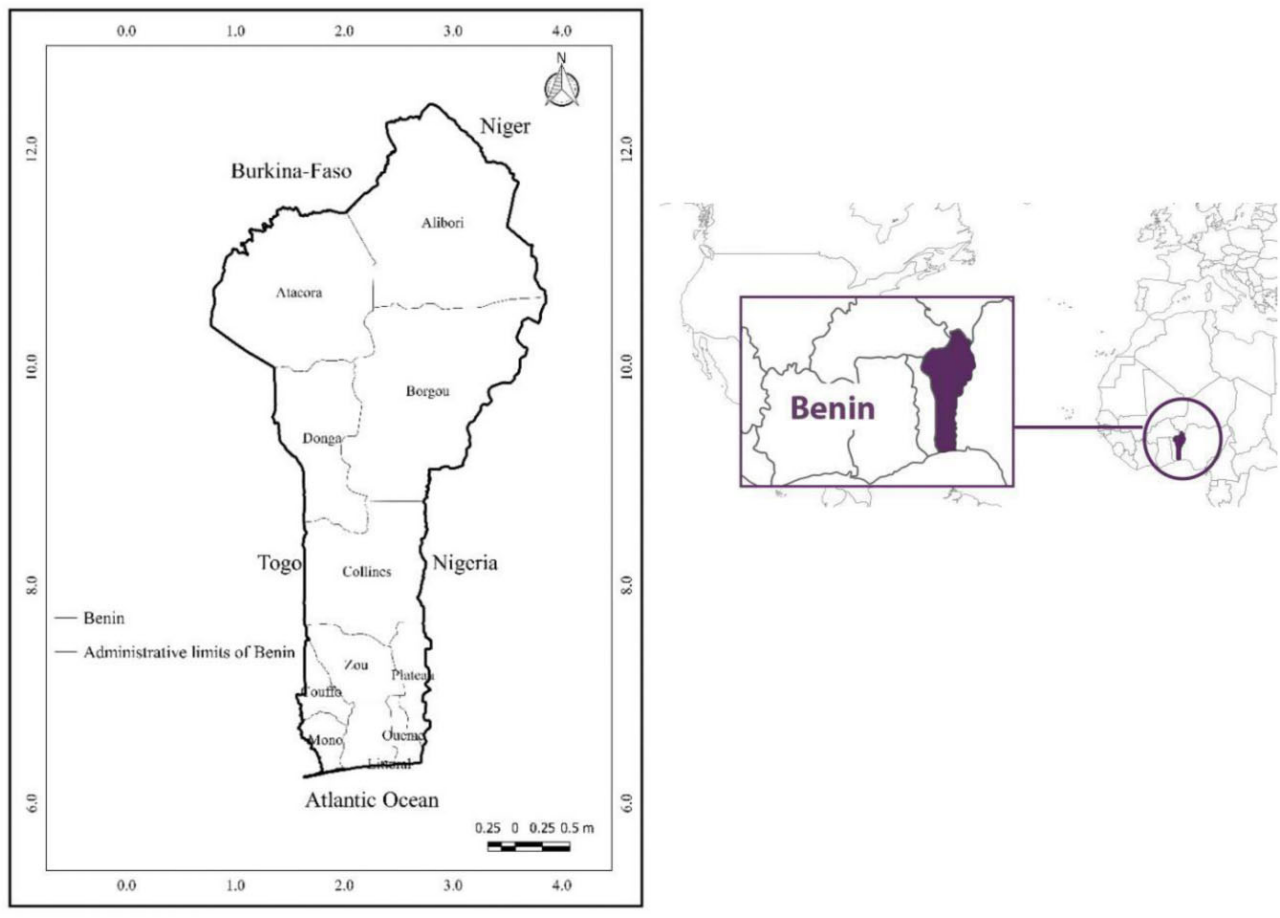
Map of the Republic of Benin.

This study was conducted in Benin, a West African country located in the intertropical zone between latitudes 6°15' and 12°25' north, and longitudes 0°40' and 3°45' east, covering a total area of 114 763 km^2^.^21^ Benin is bordered by Burkina Faso to the north, the Atlantic Ocean to the south, Togo to the west and Nigeria to the east (Figure 1). In the southern regions, average monthly temperatures range from 26 to 28°C, while in the north, temperatures often exceed 35°C, reaching up to 40°C in areas like Kandi.^22^ Annual rainfall varies between 900 and 1400 mm, supporting diverse vegetation types from shrubby bushlands in the south to open forests and savannahs in the north.^23^

As of 2024, Benin's population was estimated at 14.1 million,^23^ with around 60% of the population engaged in agriculture, fish farming and hunting.^24,25^ Malaria remains endemic in the country, placing the entire population at risk.^26^ The diverse climatic conditions across Benin have a direct impact on the spatial distribution of malaria vectors, transmission dynamics and the overall vulnerability of its inhabitants to the disease.

### Occurrences data

Occurrence data for *A. gambiae* were retrieved from the Global Biodiversity Information Facility (GBIF).^27–29^ The dataset includes presence records across Benin, compiled from various entomological surveys and specimen collections. These surveys span from 1990 to 2024, with georeferenced entries distributed across both rural and urban areas of Benin.

Because of the nature of the GBIF as an aggregation platform, detailed metadata about the individual sampling protocols were not consistently available. However, the records originate from recognized institutions and published studies, including museum specimens and vector monitoring efforts.

### Environmental variables

Environmental data play a crucial role in determining species distribution. In this study, two types of environmental variables were used: (i) bioclimatic variables representing current and future climatic conditions; and (ii) land cover/land use data. Climatic data were obtained from WorldClim version 2.1 (developed by the University of California, Berkeley, CA, USA) for current conditions (baseline: 1970–2000) and AfriClim for 2055 projections under RCP 4.5 and RCP 8.5. These datasets provide 19 bioclimatic variables at approximately 5 km resolution (2.5 arc-minutes). Initially, all 19 bioclimatic variables were considered. Landcover data were retrieved from SEDAC's^30^ Global Land Cover by National Mapping Organizations (v3) dataset, which classifies surface cover types at the same spatial resolution. This variable was included in the Maximum Entropy model version 3.4.4 (developed by the American Museum of Natural History, New York, NY, USA) as a categorical raster layer.

The future projections from AfriClim are specifically tailored to the ecological context of Africa, providing more accurate insights for the continent.^31^ The RCP 4.5 scenario represents a future where sustained mitigation efforts by governments and populations help to limit greenhouse gas emissions. By contrast, the RCP 8.5 scenario reflects a future with minimal mitigation, leading to higher emissions and more severe climate impacts. These scenarios help model the potential range shifts of species under different climate futures.^32^

### Modeling procedure and data analysis

The Maximum Entropy (Maxent)^33^ algorithm was employed using R software (V4.1.2)^34^ to model the ecological niche of *A. gambiae*. Maxent, known for its high performance, predicts potential species distribution using presence-only data combined with environmental layers.^35,36^ This method is particularly effective in approximating a species’ realized distribution.^35^

To address multicollinearity, variance inflation factors (VIFs) were calculated in R, allowing for the selection of the least correlated variables, thus improving model performance. Based on a VIF threshold of 10, we retained the following five non-collinear variables: Bio3–Isothermality; Bio4–Temperature Seasonality; Bio13–Precipitation of Wettest Month; Bio16–Precipitation of Wettest Quarter; and Landcover. All environmental layers were resampled and clipped using QGIS 3.40.5^37^ to match the Benin study area.

Occurrence data for *A. gambiae*, sourced from the GBIF, were thoroughly cleaned, removing records without geographic coordinates or those located outside of Benin.^29^ Duplicate records were also removed using the spThin package,^38^ ensuring only one occurrence per 5 km × 5 km grid.

For model calibration, the default settings were applied,^39^ with 10 000 background points generated randomly. A cross-validation method with two repetitions was used. The dismo and raster packages were employed for the analysis.^40^ The model's performance was evaluated using two statistical tests: area under the receiver operating characteristic curve (AUC) and true skill statistic (TSS).^33,41^ AUC measures the model's discriminatory ability between presence (y=1) and absence (y=0) sites by plotting sensitivity against specificity^30^; TSS, which ranges from −1 to 1, gauges predictive accuracy, with values closer to 1 indicating better performance in distinguishing between presence and absence points.

Finally, the contribution of environmental variables to identify suitable habitats for *A. gambiae* was assessed using the jackknife test of variable importance and analysis of each variable's percentage contribution.^42^

## Results

### Occurrence data of *A. gambiae* from the GBIF database

**Figure 2.**
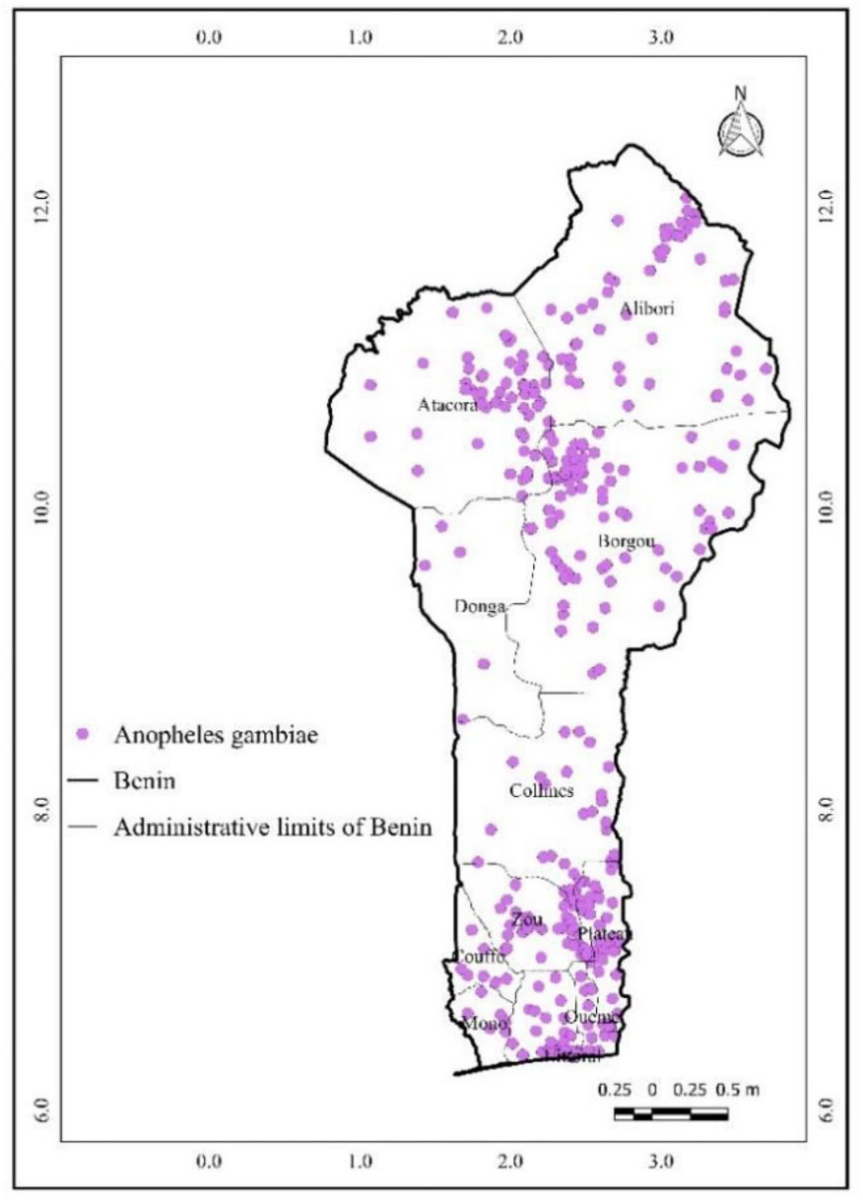
*Anopheles gambiae* occurrence data from the GBIF. GBIF: Global Biodiversity Information Facility.

A total of 4247 occurrence records of *A. gambiae* were obtained from the GBIF across various regions of Benin (Figure 2). These data serve as a crucial foundation for understanding the distribution patterns of this malaria vector within the country.

### Model performance and result interpretation

Similar to clinical studies in the health sector that evaluate the accuracy of disease diagnoses, many ENM studies utilize sensitivity and specificity curves, commonly known as receiver operating characteristics curves, to assess model relevance.^43^

Table 1 summarizes the statistical tests performed and their corresponding results, providing a comprehensive evaluation of model performance. Three key tests were conducted: the AUC, the TSS and the correlation coefficient (COR). Collectively, these values highlight the robustness of the models. The AUC, which offers a threshold-independent assessment of model performance, ranges from 0 to 1, with higher values indicating better accuracy. An AUC score of 0.75 signifies strong predictive capability. The TSS evaluates the predictive accuracy of species distribution models, with values ranging from −1 to 1. A TSS value of 0.59 reflects the model's effective ability to differentiate between presence and absence data. Finally, the COR test measures the correlation between predicted presence, absence and pseudo-absence points with actual occurrence data. A COR value of 0.57 indicates a solid relationship between predictions and real-world data.

**Table 1.** Results of statistical tests for model evaluation

Method	AUC	TSS	COR
Maxent*	0.75	0.59	0.57

AUC: area under the receiver operating characteristic curve; COR: correlation coefficient; Maxent: Maximum Entropy; TSS: true skill statistic.

*Metrics were computed based on the testing subset of data (20%) using 10-fold cross-validation.

A 10-fold cross-validation procedure was applied to partition the data into training (80%) and testing (20%) sets. All performance metrics reported here were calculated on the testing data. The AUC value of 0.75 indicates moderate ability of the model to discriminate between presence and background points. Similarly, the COR value of 0.57 reflects moderate predictive accuracy. The TSS score of 0.59, which takes into account both sensitivity and specificity, further supports a moderate level of model performance. While these values suggest that the model provides useful predictions, we acknowledge that there is room for improvement, and future work should explore ensemble approaches and incorporate additional covariates to enhance accuracy.

The continuous output from Maxent represents the habitat suitability for *A. gambiae*, ranging from 0 (unsuitable) to 1 (highly suitable). To improve interpretability for public health planning, we reclassified the suitability values into three risk levels using the natural breaks (Jenks) classification method in QGIS: low risk: 0.00–0.33; medium risk: 0.34–0.66; and high risk: 0.67–1.00.

This classification was applied consistently to current and future projection maps. While this approach introduces some simplification, it facilitates clearer communication of priority areas for malaria surveillance and intervention.

### Variable contributions to the models

**Figure 3.**
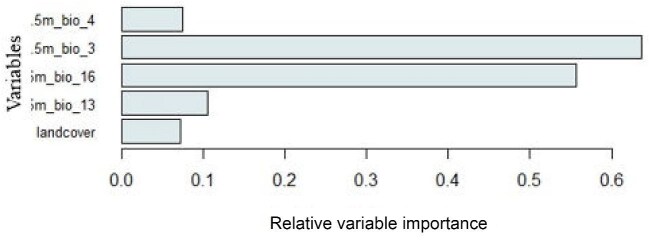
Contribution of variables to the models.

Figure 3 illustrates the relative importance of variables in the model predicting the potential distribution of *A. gambiae*. Five key variables were identified as contributing significantly: Isothermality (bio3), Temperature Seasonality (bio4), Precipitation of the Wettest Month (bio13), Precipitation of the Wettest Quarter (bio16) and Landcover. Among these, Isothermality (bio3) emerged as the most influential factor, followed by Precipitation of the Wettest Quarter (bio16), Precipitation of the Wettest Month (bio13), Temperature Seasonality (bio4) and, finally, Landcover. This ranking underscores the critical role of climatic factors in shaping the distribution of *A. gambiae*.

### Current spatial distribution of *A. gambiae* in Benin

**Figure 4.**
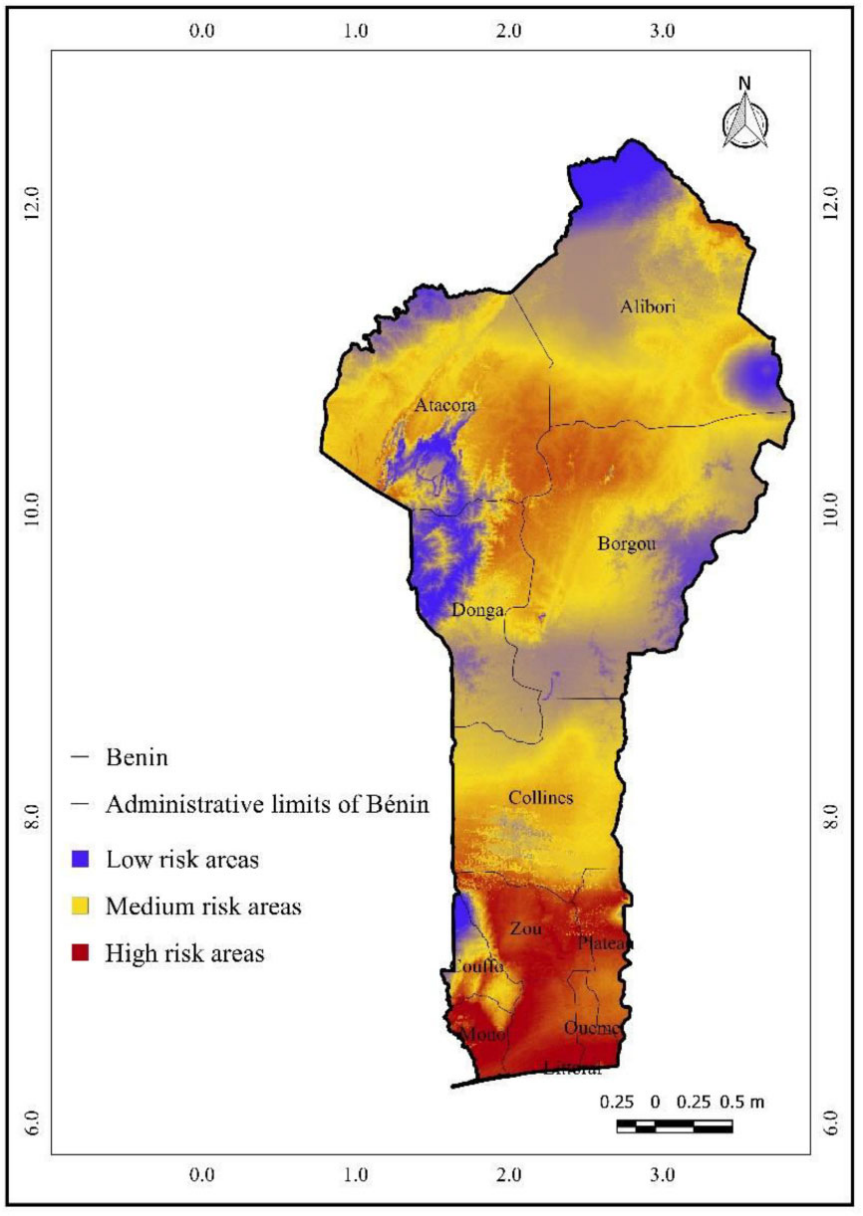
Predicted current spatial distribution of *Anopheles gambiae* in Benin based on Maximum Entropy modeling.* *Risk levels (low, medium, high) are derived from continuous suitability scores using the natural breaks (Jenks) classification method.

Figure 4 illustrates the projected spatial distribution of *A. gambiae* across Benin in the present time. The high-risk areas (depicted in red) are more localized in the extreme southern regions, including the Mono and Couffo localities and the southernmost part of Ouémé department. The medium-risk areas (in yellow) dominate central Benin but seem to occupy a slightly larger area, particularly in the northern part of the Borgou and Donga regions, and also extend further into the Atacora and Alibori regions. The low-risk zones are also primarily in the north but are slightly more fragmented, with pockets of blue appearing within the yellow medium-risk areas, particularly in Atacora and Borgou.

### Future spatial distribution projections of *A. gambiae* under RCP 4.5

**Figure 5.**
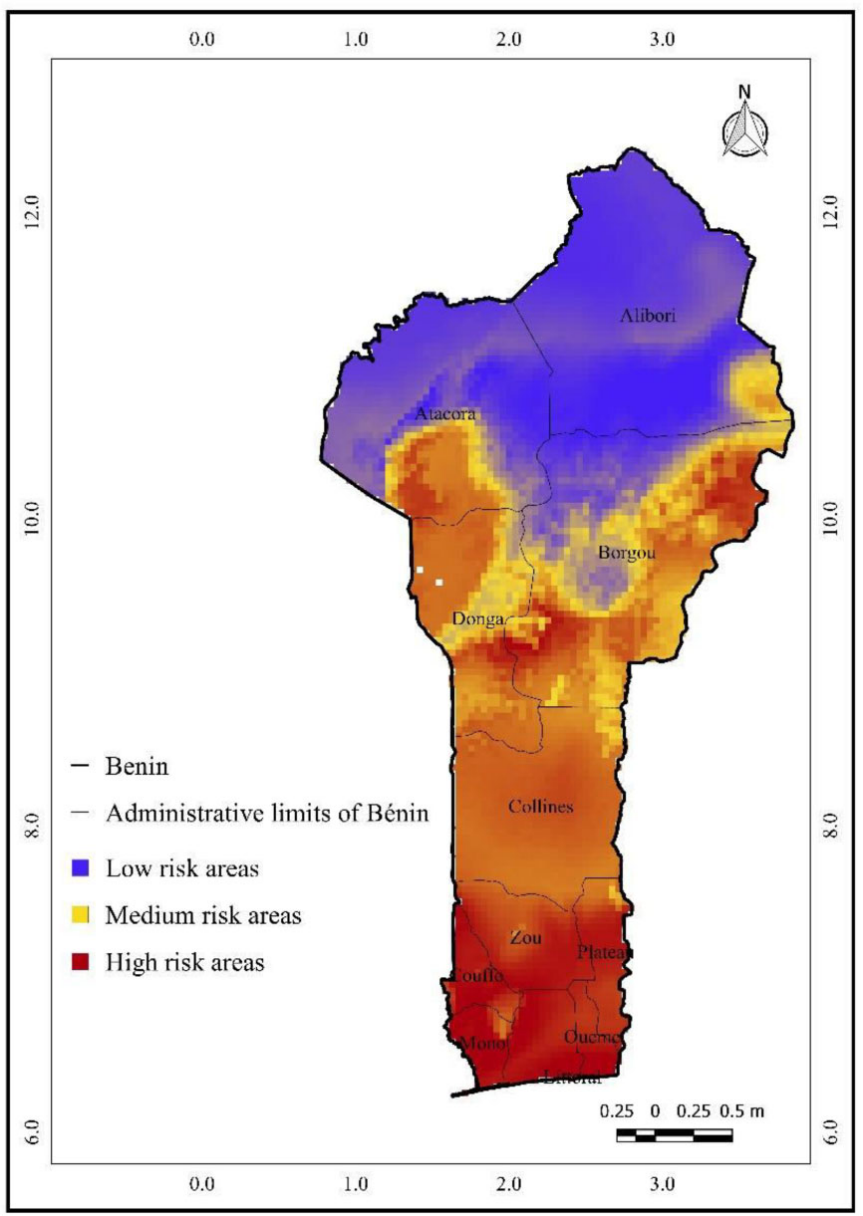
Projected spatial distribution of *Anopheles gambiae* by 2055 under the RCP 4.5 scenario.

Figure 5 projects the future spatial distribution of *A. gambiae* in Benin by 2055, under the RCP 4.5 climate scenario. High-risk areas are concentrated in the southern part of Benin, covering regions such as Zou, Plateau and Couffo. The red zones also extend further into Donga in the central-west region. Medium-risk areas cover a significant portion of central Benin, extending from Donga and Borgou down to Collines and parts of Zou. This map shows a more continuous stretch of yellow across central Benin. The low-risk areas are predominantly located in the northern regions of Benin, particularly in Alibori and Atacora, with a clear delineation between the blue and yellow zones. This forecast underscores the potential future shift in malaria risk zones, emphasizing the importance of proactive mitigation strategies.

### Future spatial distribution projections of *A. gambiae* under RCP 8.5

**Figure 6.**
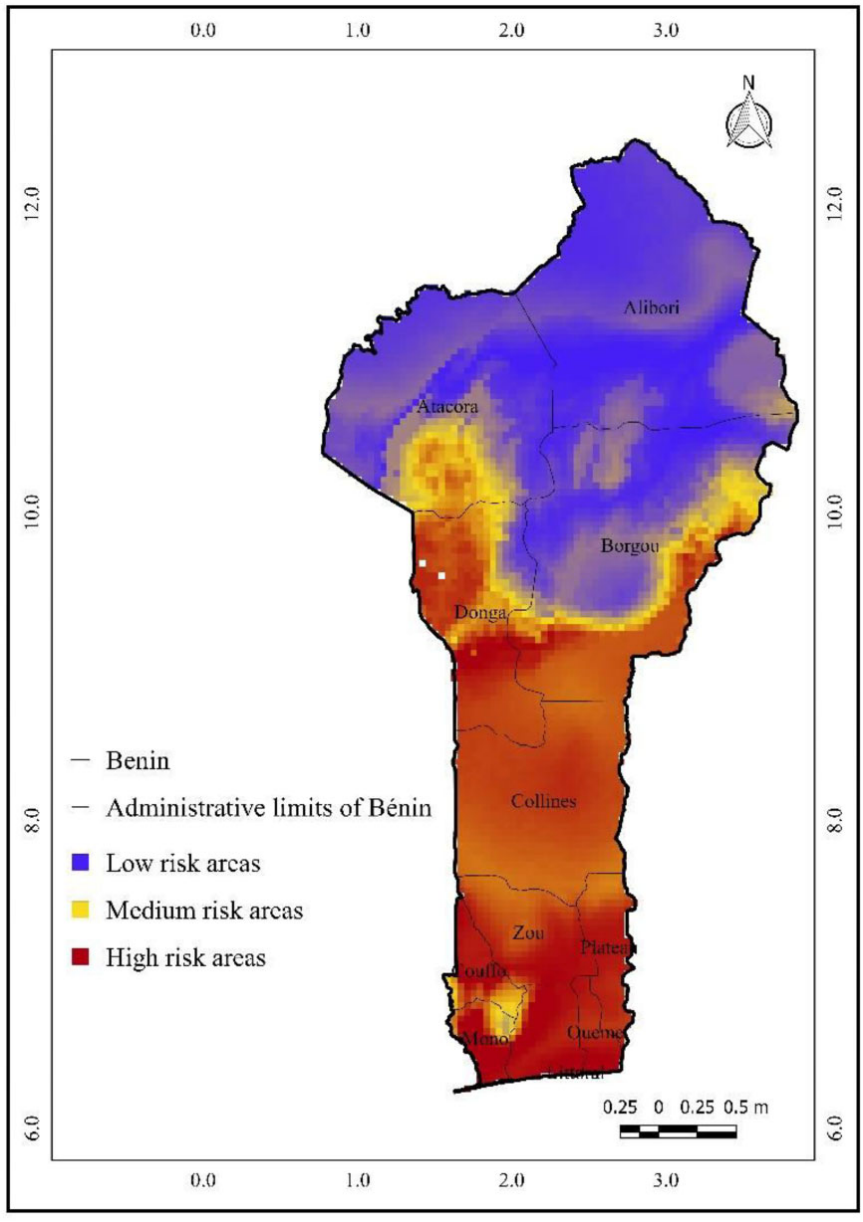
Projected spatial distribution of *Anopheles gambiae* by 2055 under the RCP 8.5 scenario.

Figure 6 presents the projected spatial distribution of *A. gambiae* by 2055 under the RCP 8.5 scenario for Benin. In this more severe climate change scenario, high-risk zones for malaria transmission (marked in red) are expected to dominate the departments of Mono, Littoral, Couffo, Ouémé, Plateau, Zou and Collines and parts of Donga, Borgou and Atacora. Medium-risk areas (in yellow) are projected to shrink, covering only small sections of Collines, Donga, Borgou and Atacora. Low-risk zones (in blue) are forecasted to encompass the entire Alibori department, along with significant parts of Borgou and smaller regions of Donga and Atacora. This highlights the intensified spread of high-risk areas under the RCP 8.5 scenario, indicating a pressing need for adaptive strategies to mitigate future malaria risks.

## Discussion

### Climate change and malaria vector dynamics

Climate change can significantly alter the distribution of pathogens, vectors and hosts, leading to shifts in disease transmission patterns. Several comprehensive studies have highlighted the profound influence of climate change on the frequency of infectious diseases by disrupting the delicate interactions among vectors, pathogens, hosts and their environments.^44,45^ Patz et al.^44^ examined how variations in temperature, humidity and precipitation influence the spread of vector-borne diseases, emphasizing the importance of understanding these dynamics to formulate effective prevention and adaptation strategies. Similarly, McMichael et al.^45^ explored how climate change impacts global health, with a particular focus on increased infectious disease risk due to altered ecosystems and vector life cycles. Other studies have further documented the critical role of climate in the development of disease vectors, particularly Anopheles mosquitoes, which are responsible for malaria transmission.^46,47^ Swaroop's early work^47^ demonstrated how temperature, humidity and precipitation affect the life cycle of Anopheles mosquitoes, while Mabaso et al.^48^ affirmed the dependence of malaria transmission on the availability of liquid water and suitable temperatures for mosquito development. Several additional studies, including those by Peterson^49^ and Siraj et al.,^50^ have underscored the correlation between climate conditions and the distribution of Anopheles species.

### Environmental drivers of suitability

Among the environmental variables included in our model, Bio3 (Isothermality) and Bio16 (Precipitation of the Wettest Quarter) had the highest contribution to habitat suitability. Isothermality (Bio3) reflects the ratio of the mean diurnal temperature range to the annual temperature range, providing a measure of temperature stability. High isothermality values indicate relatively stable temperatures between day and night, which can enhance mosquito survival rates and reproductive efficiency. *Anopheles gambiae* is known to thrive in environments with consistent temperature ranges, as extreme fluctuations can disrupt their life cycle stages, particularly egg and larval development.^50–55^ Precipitation of the Wettest Quarter (Bio16) serves as a proxy for the availability of aquatic habitats suitable for mosquito breeding. During periods of heavy rainfall, stagnant water accumulates in puddles, ditches and manmade containers, providing ideal sites for oviposition and larval development. This aligns with previous findings that *A. gambiae* populations surge during rainy seasons in sub-Saharan Africa.^48^ The model's sensitivity to Bio16 highlights the reliance of this species on seasonal water availability, reinforcing the need for enhanced larval source management during peak rainfall months.

### Spatial trends and future risk

Our results indicate that current high-risk areas for malaria in Benin are concentrated in the southern coastal regions (Mono, Littoral, Couffo, Ouémé, Plateau and Zou) and parts of central Benin (Collines, Donga and Borgou). Future projections for 2055 under both RCP 4.5 and RCP 8.5 scenarios show a continued high suitability in the southern and central regions, while potential contractions are observed in the north, notably in Alibori and Atacora. This reduction in northern suitability may be explained by projected climatic changes—particularly increased temperature extremes and drying trends—that exceed the thermal or moisture thresholds for *A. gambiae* survival and reproduction.^49,50^ These results are consistent with other modeling efforts in the Sahel region predicting a northward contraction of vector suitability under high-emission climate pathways.^56,57^ While southern areas may become more vulnerable, the northern regions may paradoxically experience decreased risk, although this could be offset by local ecological or anthropogenic factors not captured in our model.

Our findings align with those of Damien et al.^58^ and Govoetchan et al.,^59^ who observed that malaria occurs across nearly all departments in Benin. The comparison of current and projected distribution maps (Figures 4 and 5) reveals an expansion of high- and medium-risk areas under future scenarios. Under RCP 4.5, high-risk areas expand northward, and medium-risk zones cover broader regions, while low-risk zones shrink. These spatial dynamics emphasize the need for proactive planning in regions expected to transition to higher risk categories.

### Public health implications and strategic recommendations

To effectively address the future spread of *A. gambiae* and malaria in Benin, a proactive, climate-resilient approach is essential. Expanding surveillance systems to cover shifting high-risk zones will provide early warnings for emerging hotspots. Climate-adapted vector control strategies—such as insecticide-treated nets, indoor residual spraying and larval source management—should be prioritized in southern and central regions. The WHO^5^ recommends integrating climate data into public health planning, and studies by Caminade et al.,^56^ Gething et al.^57^ and Paaijmans et al.^60^ support the need for climate-informed, region-specific interventions. Equally important is fostering collaboration between meteorological, environmental and health institutions and promoting community engagement to raise awareness and support local surveillance and response efforts. Strengthening health systems, especially diagnostic capacity and rapid treatment access in vulnerable areas, will be vital to mitigate climate-related increases in malaria transmission.^61–63^

While ENM provides a valuable tool for projecting the potential distribution of malaria vectors, it has limitations. Our model focused exclusively on *A. gambiae* due to data availability and did not consider other malaria vector species. The model also excluded human-related factors such as urbanization, socioeconomic variables and public health interventions such as vaccination. Furthermore, the Maxent algorithm, while widely used for presence-only data, does not incorporate absence or pseudo-absence data as effectively as ensemble approaches such as Biomod2. We selected Maxent for its strong performance with our data type, its simplicity and its extensive use in vector modeling literature. Nonetheless, future research should integrate multiple algorithms and additional variables to refine predictions and improve model robustness.

This study serves as a foundation for integrating climate modeling into national malaria strategies and underscores the potential of ENM in informing public health decisions. Our methods can be adapted to forecast the spread of other climate-sensitive, vector-borne diseases such as dengue and yellow fever. Similar modeling approaches have successfully informed preparedness strategies for Ebola, Marburg and other emerging diseases.^20,64–67^ In this context, predictive modeling tools are increasingly essential for building resilient, climate-adaptive public health systems.

## Conclusions

This study highlights the projected shifts in the distribution of *A. gambiae*, Benin's primary malaria vector, under future climate change scenarios. Our modeling reveals that temperature stability and increased seasonal precipitation will likely expand vector habitat suitability in southern and central Benin. If left unaddressed, these changes could lead to a significant expansion of high-risk malaria zones and intensify the public health burden. These findings emphasize the need to incorporate environmental and climate data into malaria surveillance systems. Strengthening early warning systems and promoting adaptive vector control strategies will be vital in preparing for future outbreaks. The research provides a scientific basis for policy decisions aimed at protecting at-risk communities. By anticipating climate-driven disease dynamics, health authorities can design more effective and geographically targeted interventions to mitigate the spread of malaria.

## Data Availability

The data that support the findings of this study are available from the corresponding author upon reasonable request.
